# Regulation of functional corneal endothelial cells isolated from sphere colonies by Rho-associated protein kinase inhibitor

**DOI:** 10.3892/etm.2012.816

**Published:** 2012-11-19

**Authors:** YAN-LONG BI, QI ZHOU, FEI DU, MING-FENG WU, GUO-TONG XU, GUI-QIN SUI

**Affiliations:** 1 Department of Ophthalmology, Tongji Hospital affiliated with Tongji University School of Medicine; Shanghai 200065;; 2 Department of Regenerative Medicine and Stem Cell Research Center, Tongji University School of Medicine, Shanghai 200065;; 3 Department of Ophthalmology, Jilin University Bethune Second Hospital, Changchun 130041, P.R. China

**Keywords:** corneal endothelial cell, Rho-associated protein kinase signal, Y-27632, sphere-forming, Na^+^/K^+^-ATPase

## Abstract

Human corneal endothelial cells (HCECs) form a monolayer covering the inner side of the cornea. Since HCECs have lower mitotic activity after birth, damage or disease of HCECs will lead to failure of cellular pump functions and inevitably to corneal stroma oedema and loss of vision. *Ex vivo* cultured HCEC transplantation is the most promising therapy used to avoid donor tissue shortages. However, proliferation of functional adult HCECs is difficult to achieve using standard cell culture techniques. In this study, we isolated sphere colonies from HCECs, which were characterised as HCEC precursor cells. HCECs from these spheres were incubated with the Rho-associated protein kinase (ROCK) inhibitor Y-27632 (30 μM for 1 h at 37°C) to observe cell pump functions and the levels of several cell markers. Cells from the spheres were immunostained positive for nestin (a marker of immature cells) and for an immature neuronal marker (β3-tubulin). There were no significant differences in the expression of genes associated with cell adhesion or ion transport channels, between the cells with or without Y-27632. However, immunostaining revealed a higher density of Na^+^/K^+^-ATPase in cell nuclei from HCECs (sphere-forming) and HCECs (sphere-forming/Y-27632) than normal HCECs. There was a higher resting potential difference and resting short circuit in the HCEC (sphere-forming) and HCEC (sphere-forming/Y-27632) cell lines, compared with normal HCECs. In this study, we successfully cultured functional HCEC cell lines, which possessed several important characteristics required to maintain the transparency and refractive parameters of the human cornea. These included hexagonal cell morphology, higher cell adhesion and proliferation ability, HCEC pump function and positive expression of several cell adhesion markers.

## Introduction

Human corneal endothelial cells (HCECs) form a homogeneous single layer of flat hexagonal cells, which are attached to the basement membrane (Descemet’s membrane). HCECs are essential for maintaining corneal transparency, which is dependent upon endothelial regulation of stromal hydration, including the barrier and pump functions of the aqueous humor (1). Damage to HCECs caused by intraocular surgery, glaucoma, trauma or diseases, including Fuchs’ corneal dystrophy, may result in irreversible corneal edema, since there is little or no mitotic activity in the HCECs after birth (2). Despite several successful, recently developed HCEC replacement surgeries, including Descemet’s membrane endothelial keratoplasty (DMEK) and Descemet’s stripping automated endothelial keratoplasty (DSAEK), owing to the aging population, increased requirements for corneal transplants and an insufficient quantity of available donor tissues has led researchers to explore alternative ways of preparing *in vitro* corneal endothelial cell monolayers (3). However, proliferation of functional adult HCECs is difficult to achieve using standard cell culture techniques (4). HCECs were originally believed to be incapable of dividing *in vitro*, but have been successfully isolated and cultured with epidermal growth factor (EGF), platelet-derived growth factor (PDGF), bovine pituitary extract and fetal bovine serum (FBS). However, the number of cells with proliferative activity and the ability to respond to such agents is relatively low and much variation in morphology and function exists after several cell passages (4–6). Thus, there is a need to develop a stable and effective cell culture system to maximize cell proliferation and maintain physiological function. In this study, we successfully developed a HCEC cell culture system which maintains cell functions.

## Materials and methods

### Primary HCEC culture

Human donor corneas were obtained according to the principles set out in the Declaration of Helsinki. Whole donor corneas (n=6) with a corneal endothelial cell density of 1500–2000/mm^2^ and corneal scleral rims (n=6), following penetrating keratoplasty, were obtained from the Tongji University Hospital eye bank (Shanghai, China), following ethical approval and specific consent for research use. The mean donor age was 67.2±11.1 and ranged from 56 to 76 years. Under a dissecting microscope, the Descemet’s membrane with the endothelium attached was carefully peeled off. The removed membrane was cut into small pieces, ∼1–2 mm in diameter and cultured in a cell culture dish covered with type IV collagen in Dulbecco’s modified Eagle’s medium [DMEM/nutrient mixture F12 (1:1)] with high glucose, supplemented with 15% FBS, insulin-transferrin-selenium and minimal essential medium-non-essential amino acid (MEM-NEAA; Gibco, Carlsbad, CA, USA). Cells were subcultured after reaching confluency by treating with trypsin/ethylenediaminetetraacetic acid (EDTA) and then seeded at a density of 5×10^5^ cells/well in 6-well dishes. Second or third passage cells were used for further experiments.

### HCEC sphere-forming cultures

Cell culturing followed the method previously reported (7) with certain modifications. Briefly, cells were plated in 24-well super hydrophilic plates at densities ranging from 1×10^4^ to 1×10^5^ cells/plate, then further cultured in DMEM/F12 (1:1; Gibco) supplemented with B27 (Invitrogen, Carlsbad, CA, USA), 40 ng/ml basic fibroblast growth factor (bFGF; R&D Systems, Minneapolis, MN, USA), 20 ng/ml EGF (Sigma-Aldrich, St. Louis, MO, USA) and N2 supplement (Gibco). Basal medium containing methylcellulose gel matrix (1.5%, Wako Pure Chemical Industries, Osaka, Japan) was used to prevent the reaggregation of cells. Primary sphere-forming cells cultured for 7 days were used in these studies. For the secondary sphere formation assays, primary spheres were incubated in 0.05% trypsin and 0.02% EDTA and the dissociated cells were plated at 5×10^4^ cells/well, following the same culture method as described above.

### Treatment with Y-27632

HCECs from sphere-forming cultures were incubated with medium containing Y-27632 (30 μM, for 1 h at 37°C; Shanghai Biochempartner Co, China). The treated cells were harvested for further experiments.

### Homotypic adhesion assay

The homotypic adhesion assay was performed as reported previously (8). Briefly, monolayer HCECs, incubated as described above, on 24-well plates were gently washed three times with phosphate-buffered saline (PBS). Cells (1×10^5^) in 1 ml medium with or without Y-27632 at 30 μM were seeded into each well. The 24-well plate was then placed in a horizontal shaker and agitated at 70 rpm at 37°C. The unattached cells were removed prior to calculating cell number under a microscope, after incubation for 10, 30 and 60 min. The number of attached cells was calculated using the following formula: number of adherent cells = 1×10^5^ – number of unattached cells.

### Immunocytochemistry

Immunostaining of cells was used to localize ZO-1, Na^+^/K^+^-ATPase, β3-tubulin and nestin proteins. The primary antibodies used were: mouse anti-human ZO-1 mAb (clone 1, isotype IgG; BD Biosciences, San Jose, CA, USA; dilution 1:200 in PBS, incubation time, 45 min), mouse anti-human Na^+^/K^+^-ATPase mAb (clone 9-A5, isotype IgG1; Abcam, Cambridge, UK; 1:200, 45 min), mouse anti-nestin mAb (BD Pharmingen, San Diego, CA, USA; 1:200, 45 min) and rabbit anti-β3-tubulin polyclonal antibody (Covance Research Products, Danvers, PA, USA; 1:2000, 45 min). Following treatment with the primary antibody, 1% (w/w) bovine serum albumin (BSA) was added to the PBS solution to remove unbound antibodies. In all cases the secondary antibody was Alexa Fluor 546 goat anti-mouse IgG (γ1; Invitrogen; 1:200, 30 min). Cells were fixed with 4% paraformaldehyde in PBS for 10 min then incubated with 0.5% Triton X-100 and 1% (w/w) BSA in PBS for 10 min, prior to exposure to the primary and secondary antibodies. Following these steps, the samples were rinsed twice in PBS.

### Semi-quantitative reverse transcription-polymerase chain reaction (RT-PCR)

Total RNA was extracted from cultured HCECs using the RNeasy plus mini kit (Qiagen, Beijing, China) according to the manufacturer’s instructions and quantitated at 260 nm. Total RNA was then reverse transcribed into cDNA using Superscript III reverse transcriptase (Invitrogen) with oligo random hexamers. The cDNAs of each component were amplified by PCR using specific primers and DNA polymerase. The reaction was first incubated at 95°C for 10 min, followed by 39 cycles at 98°C for 30 sec, 58°C for 30 sec and 74°C for 30 sec. RT-PCR primers are listed as follows: Na^+^/K^+^-ATPase, forward, 5′-CCC AGG ACT CAT GGT TTT TC-3′, reverse, 5′-GGA GCA AAG CTG ACC TGA AC-3′; β-catenin, forward, 5′-TAC CTC CCA AGT CCT GTA TGA G-3′, reverse, 5′-TGA GCA GCA TCA AAC TGT GTA G-3′; ZO-1, forward, 5′-AGT CCC TTA CCT TTC GCC TGA-3′, reverse, 5′-TCT CTT AGC ATT ATG TGA GCT GC-3′.

### Flow cytometry analyses

For Ki67 studies, HCECs prepared from sphere-forming colonies with or without Y-27632 treatment were passaged in 1:4 dilutions and dissociated into single cells by 0.25% trypsin digestion, fixed in 70% (w/v) ethanol, then washed and incubated for 20 min with 1% BSA. The HCECs were incubated with a 1:20 dilution of anti-mouse Ki67, then washed and incubated with 1:1000 diluted Alexa Fluor 488 conjugated goat anti-mouse IgG (Invitrogen), according to the manufacturer’s instructions. Flow cytometric analyses were then performed using a FACSCalibur flow cytometer (BD Biosciences).

### Measurement of corneal endothelial cell pump function

The pump function of confluent monolayers of HCECs was measured using an Ussing chamber as described previously (9). Cells cultured on Snapwell inserts coated with type IV collagen were placed in the Ussing chamber with the endothelial cell surface side in contact with one chamber and the Snapwell membrane side in contact with another chamber. The chambers were carefully filled with Krebs-Ringer bicarbonate and maintained at 37°C using an attached heater. The short circuit current was measured with narrow polyethylene tubes positioned close to either side of the Snapwell insert and filled with 3 M potassium chloride (KCl) and 4% agar gel connected to silver electrodes. These electrodes were connected to the computer through the Ussing system VCC-MC2 (Physiologic Instruments, San Diego, CA, USA) and an iWorx 118 Research Grade Recorder (iWorx Systems, Dover, NH, USA). After the short circuit current had reached a steady state for 10 min, ouabain (1 mM), a Na^+^/K^+^-ATPase inhibitor, was added to the chamber and the short circuit current was re-measured.

### Statistical analysis

All experimental results were analyzed by one-way analysis of variance using SPSS version 12.0 software (SPSS Inc., Chicago, IL, USA). Summary statistics were expressed as mean ± standard deviation (SD). P<0.01 was considered to indicate a statistically significant difference and all p-values were two-sided.

## Results

### Isolation of sphere colonies from HCECs

**Figure 1.**
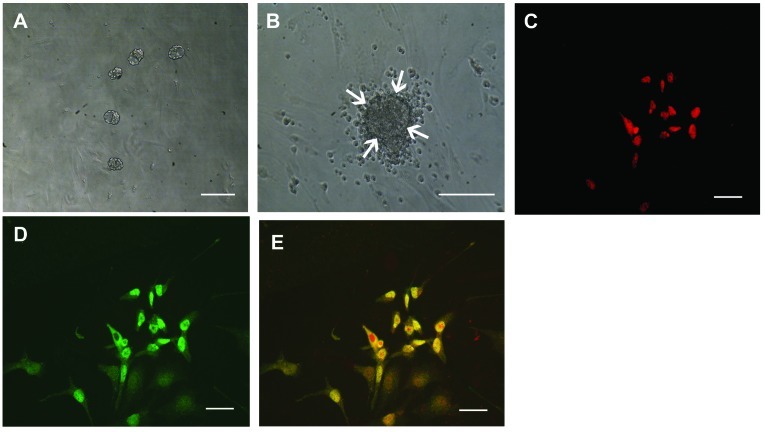
Sphere culture of human peripheral corneal endothelial cells. (A) HCEC spheres on day 7 on the surface of the fibroblast substrate. (B) Cells migrating from an attached sphere on adherent substrate on day 10. The arrowheads show the contour of the sphere. (C–E) Immunocytochemical analysis of the differentiated cells derived from spheres. The cells are double immunostained by (C) nestin and (D) β3-tubulin antibodies. (E) Merged staining. (A and B) scale bar = 100 μm. (C–E) scale bar = 50 μm. HCEC, human corneal endothelial cell.

Spheres developed from the single cell suspensions on day 7 (Fig. 1A) and grew larger by day 10 (Fig. 1B). A number of cells migrated from the sphere colonies (Fig. 1B) and the progeny from sphere colonies could be passaged twice, whereas the non-proliferating cells died. The number of spheres was counted after 10 days of culture, revealing that 51.2±8.1 spheres (mean ± SD, n=8) were generated per 10,000 cells. When the primary sphere colonies from HCECs were dissociated into single cells and were cultured in the presence of the methylcellulose gel matrix, secondary and tertiary sphere colonies were generated. This suggests that HCECs had the capacity for self-renewal of sphere colonies, although this capacity was limited. The cells released from the spheres were immunostained for nestin (as a marker of immature cells, Fig. 1C) and also immunostained positive for an immature neuronal marker, β3-tubulin (Fig. 1D).

### Effects of Y-27632 on adhesion capacity and cell cycle progression of HCECs

**Figure 2.**
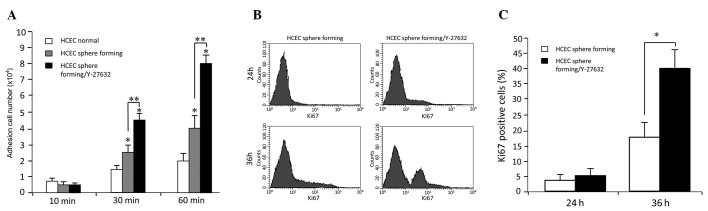
Effects of Y-27632 on adhesion of HCECs. (A) HCECs were treated with 30 μM Y-27632 for 1 h. The number of adhesion cells from three independently repeated experiments are expressed as mean ± standard deviation (SD) in homotypic adhesion assays. ^*^P<0.01, compared with normal HCECs; ^**^P<0.01, compared with sphere-forming HCECs. (B) Ki67-positive cells were analyzed by flow cytometry. HCECs were subcultured for 36 h and stained with Ki67 antibody. (C) The number of Ki67-positive cells was significantly elevated in the presence of Y-27632 at 36 h (^*^P<0.01). Data are expressed as the mean ± SD (n=6). HCEC, human corneal endothelial cell.

To investigate the effect of the Rho-associated protein kinase (ROCK) signalling pathway on adhesion of HCECs, a homotypic adhesion assay was performed. There were statistical differences between the untreated cells and cells treated with 30 μM Y-27632, after 30- and 60-min incubations (p<0.05), however not at 10 min. The increase of adhesion cells was time-dependent (Fig. 2A).

Quantitative flow cytometric analysis revealed a significantly higher presence of Ki67-positive cells in HCECs cultured with Y-27632, 36 h after subculture (Fig. 2B and C), thus demonstrating that Y-27632 alters HCEC proliferation.

### Effects of Y-27632 on gene expression and density of Na^+^/K^+^-ATPase-positive cells

**Figure 3.**
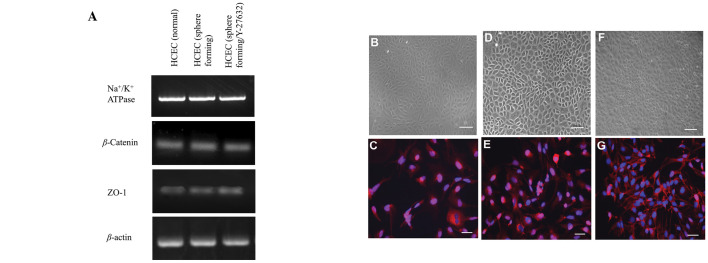
HCEC-associated genes and cell localization of junctional components expressed by cell lines. (A) Semi-quantitative reverse transcription-polymerase chain reaction for HCEC-associated genes. Total RNA was prepared from cultured cells 7 days after reaching confluency. No significant difference in mRNA expression was observed among the three cell lines. Compared with phase contrast micrographs (B, D and F), immunostaining demonstrated that Na^+^/K^+^-ATPase was localized between cell nuclei (blue, nuclei; red, Na^+^/K^+^-ATPase) with differences shown in the three cell lines (C, E and G). B, D and F, scale bar = 100 μm; C, E and G, scale bar = 50 μm. HCEC, human corneal endothelial cell.

Expression of genes involved in active transmembrane transporter activity (Na^+^/K^+^-ATPase) or cell adhesion (ZO-1 and β-catenin), were assessed by semi-quantitative RT-PCR (Fig. 3A). No significant difference was observed among the three cell lines in the expression of genes associated with several cell adhesion or ion transporter channel proteins, which are characteristically expressed by HCECs. Na^+^/K^+^-ATPase, a key HCEC transmembrane protein, demonstrated positive staining at the intercellular junction in HCECs (Fig. 3C, E and G). Although hexagonal morphology was identified by phase contrast microscopy and immunocytochemistry, differences among the three cell lines were not observed. Immunostaining revealed a high density of Na^+^/K^+^-ATPase located between cell nuclei in the HCECs (sphere-forming) and HCECs (sphere-forming/Y-27632), however not in HCECs (normal; Fig. 3C, E and G).

### Effects of Y-27632 on potential difference and short circuit current driven by Na^+^/K^+^-ATPase

**Figure 4.**
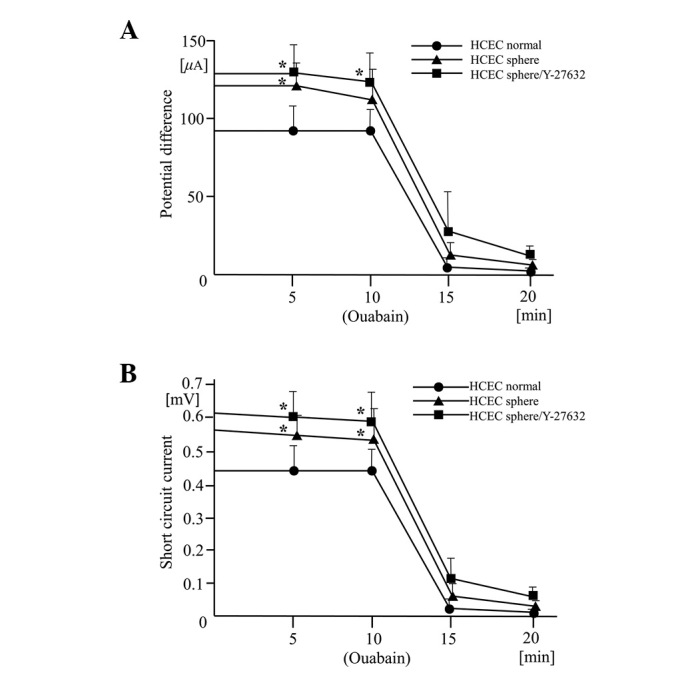
Na^+^/K^+^-ATPase activity representing the pump function of cell lines from corneal cell monolayers on the insert well area of 4.67 cm^2^. Potential difference (A) and short circuit current (B) were measured before and after adding the Na^+^/K^+^-ATPase inhibitor ouabain. ^*^P<0.01, compared with HCEC (normal). HCEC, human corneal endothelial cell.

The traces of the potential difference and short circuit current driven by the Na^+^/K^+^-ATPase were of similar shapes in the three cell lines compared with normal HCECs. However, there were higher resting potential differences and resting short circuit measurements in the HCEC sphere and HCEC sphere/Y-27632 cell lines (Fig. 4A and B). The potential difference and short circuit currents maintain corneal transparency and levels in all the cell lines were clearly reduced by the presence of the Na^+^/K^+^-ATPase inhibitor, ouabain. This confirms that the origin of the current is Na^+^/K^+^-ATPase. The pump function in normal HCECs, detected in earlier and later passages of cells, was more variable than in the other two cell lines. This possibly indicates incomplete Na^+^/K^+^-ATPase activity or the presence of an intercellular barrier that regulates ion permeability.

## Discussion

A necessary prerequisite for successful tissue engineering of the human corneal endothelium is to ensure effective isolation, preservation and expansion from a small number of HCECs (4,6). The present study revealed that spheres derived from HCECs demonstrate a high proliferative capacity and cells directly derived from the spheres expressed markers of neural (β3-tubulin and nestin) lineages. We were not able to demonstrate directly that isolated spheres gave rise to HCECs due to lack of specific markers (10). However, the characteristic hexagonal morphology and transport activity of HCECs in the Ussing chamber system suggests that the cultures largely give rise to cells that have features of HCECs (11). These findings indicate that HCEC-derived spheres have the characteristics of HCEC precursor cells.

ROCK is one of the main downstream effectors of the Ras-homologous (Rho) family of GTPases, which are involved in a number of cellular functions, including cell proliferation, apoptosis, invasion and metastasis (12). Overexpression of ROCK promotes invasion and metastasis in a number of solid tumors, including liver, breast and colon cancers (13). Although the HCECs tend to have a cell senescence phenotype after only a few passages, we successfully cultivated and passaged HCEC spheres with or without the presence of the ROCK inhibitor Y-27632. We further demonstrated elevated cell-cell adhesion by the homotypic adhesion assay and the adhesion of HCECs was dose- and time-dependent with Y-27632. Quantitative flow cytometric analyses revealed the increased presence of Ki67-positive cells in HCECs cultured with Y-27632 for only 36 h. This suggests that Y-27632 promotes HCEC proliferation. Although further studies are required, the increased cell adhesion and motility observed in our study, which was enhanced by ROCK inhibition, may also have a positive effect on HCEC proliferation. Further investigations are necessary to determine whether the increase in cell proliferation observed in our study may be attributed to an effect on the molecular components regulating cell-cycle progression (14).

Corneal endothelial cells accumulate Na^+^/K^+^-ATPase at intercellular contacts along the lateral cell membrane to maintain a bicarbonate gradient across the cell layer to sustain a constant flow of water out of the stroma (11). We detected Na^+^/K^+^-ATPase in the cells and at the lateral cell contacts. The presence of this protein in our HCEC populations indicates that HCEC cells from direct HCEC culture and from sphere-forming culture have this pump function. However, the number of Na^+^/K^+^-ATPase-positive cells was significantly higher in the HCECs (sphere-forming/Y-27632) than those observed in HCECs (sphere-forming) and HCECs (normal culture). We used the Ussing chamber assay to detect the cell pump function evaluated from cell electrophysiological measurements. Prior to the addition of the Na^+^/K^+^-ATPase inhibitor ouabain, potential difference and short circuit current were detected in the three cell lines. Compared with the values of the normal HCECs, HCEC (sphere) and HCEC (sphere/Y-27632) had a higher potential difference and higher short circuit current, which may have been caused by increased Na^+^/K^+^-ATPase activity. These results are consistent with the observation that relatively mature intercellular adhesion allows regular intercellular ion transport with differences in cellular density (11,15).

The results of this study demonstrated that Y-27632 promoted the HCEC adhesion and proliferation ability and increased the HCEC cell pump function. In addition, it was demonstrated that Y-27632 does not alter important morphological features of HCECs. These are all important characteristics required for *ex vivo* cultured HCEC transplantation.
